# A Greener Acid-free Microwave-Assisted Digestion Method for Multielement Analysis of Fish Tissues by ICP–MS

**DOI:** 10.1021/acsomega.6c08049

**Published:** 2026-10-19

**Authors:** Marcos V. S. Pereira, Elisânia K. B. Fonseca, Jemmyson R. de Jesus, Marco A. Z. Arruda

**Affiliations:** † Research Laboratory in Bionanomaterials, LPbio, Department of Chemistry, Federal University of Viçosa - UFV 36570-900, Viçosa, Minas Gerais, Brazil; ‡ Group of Spectrometry, Sample Preparation and Mechanization, GEPAM, Department of Analytical Chemistry, Institute of Chemistry, University of Campinas - UNICAMP 13083-970, Campinas, São Paulo, Brazil; § National Institute of Science and Technology for Bioanalytics-Lauro Kubota, INCTBio-LK 13083970, Campinas, São Paulo, Brazil

## Abstract

Trace element monitoring in fish tissues requires reliable and sustainable sample preparation methods, particularly for complex biological matrices. In this study, an acid-free microwave-assisted digestion (AFMAD) method based exclusively on H_2_O_2_ was developed and optimized for multielement determination in fish tissues by ICP–MS. A central composite design coupled with response surface methodology was employed to evaluate the influence of H_2_O_2_ concentration, ramp time, and digestion temperature on matrix decomposition efficiency using 100 mg of dry fish tissue subsamples throughout the method development. The optimized conditions consisted of 30% (v/v)­H_2_O_2_, 8 min ramp time, a 20 min hold time and 240 °C, resulting in residual carbon contents below 20%, which indicates efficient oxidation of the organic matrix using H_2_O_2_ in the absence of HNO_3_. The analytical performance of the proposed method was evaluated using cod muscle certified reference material (BCR-422), providing recoveries ranging from 76 to 101% for Fe, Cu, Zn, Mn, As, and Cd. Excellent linearity was obtained for all analytes (*R* ^2^ > 0.990) within the 0–50 μg L^–1^ range. Limits of detection ranged from 0.0004 to 0.04 μg g^–1^, demonstrating suitable sensitivity for trace elemental analysis in fish matrices. The applicability of the method was demonstrated through the analysis of five commercial fish species collected in the Mariana-MG region. Concentrations of toxic elements were found in *Megaleporinus conirostris* (Cd, 0.003 mg kg^–1^) and *Cichla ocellaris* (As, 0.07 mg kg^–1^) and remained below the maximum limits established by Brazilian legislation. The proposed AFMAD method demonstrated satisfactory analytical performance for complex fish matrices while eliminating the use of concentrated mineral acids, reducing hazardous reagent consumption, and improving the sustainability characteristics of sample preparation procedures for elemental analysis.

## Graphical abstract

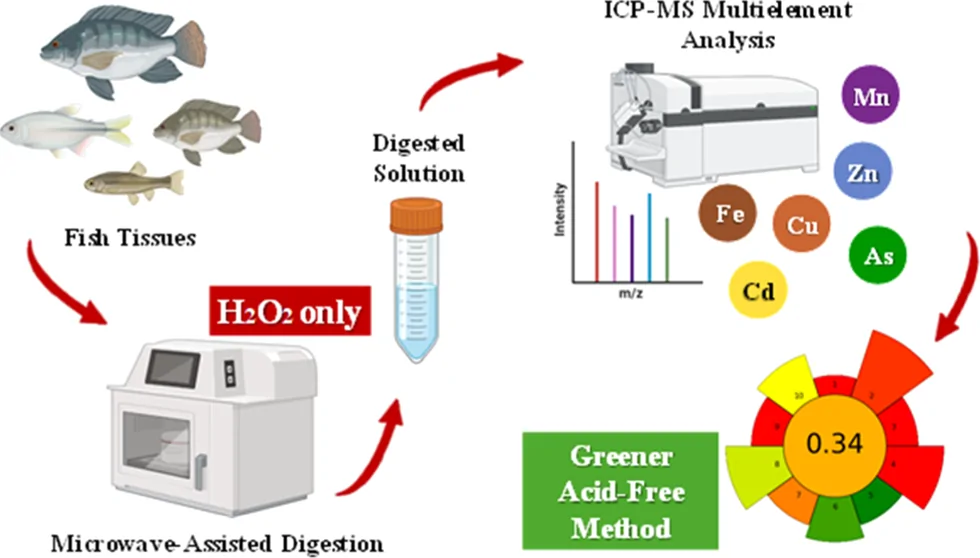

## 1 Introduction

Mining activities play a fundamental role in economic development, however, intensive mineral exploitation may significantly increase the release of potentially toxic elements into aquatic ecosystems.^1^ In Brazil, the environmental disasters associated with the collapse of tailings dams in Mariana (November 5, 2015) and Brumadinho (January 25, 2019) highlighted the vulnerability of mining regions and raised long-term concerns regarding contamination of aquatic environments and food safety.^2^ The deposition and persistence of metal-rich residues in river basins may promote the accumulation of trace elements in aquatic organisms, particularly fish species consumed by local populations.^3^

Several trace elements naturally occur in biological systems and play important physiological roles. However, excessive accumulation may cause toxic effects and compromise ecological and human health.^4,5^ Elements such as Cd and As are especially concerning due to their high toxicity and bioaccumulative potential, even at low concentrations.^6^ Therefore, reliable analytical methodologies are essential for monitoring elemental composition in environmental and food matrices.

Inductively coupled plasma mass spectrometry (ICP–MS) is widely employed for multielement determination because of its high sensitivity, low detection limits, and capability for trace and ultratrace analysis.^7–9^ Despite its excellent analytical performance, sample preparation remains one of the most critical steps for elemental analysis, particularly for complex biological matrices. Conventional microwave-assisted digestion (MAD) commonly relies on concentrated mineral acids, especially HNO_3_, to promote matrix decomposition and analyte release.^10,11^ Although highly effective, these procedures generate hazardous residues, increase operational risks, and contribute to the environmental impact associated with analytical laboratories.

In this context, Green Analytical Chemistry has stimulated the development of more sustainable sample preparation strategies aimed at reducing reagent consumption and minimizing hazardous waste generation without compromising analytical performance.^12^ Among the proposed alternatives, acid-free digestion approaches based on hydrogen peroxide have attracted increasing attention. Under microwave irradiation and elevated temperatures, H_2_O_2_ decomposes into highly reactive oxygen species capable of oxidizing organic matter, allowing matrix decomposition without the use of concentrated mineral acids.^13^

Recent studies demonstrated the feasibility of acid-free microwave-assisted digestion for relatively simple biological matrices, such as serum, urine, and plant materials.^14^ However, the application of these strategies to complex solid biological tissues remains challenging. Fish muscle is a complex biological matrix containing substantial amounts of proteins and lipids, which may require strong oxidative conditions to achieve efficient decomposition under acid-free conditions.^15^ Incomplete oxidation may result in residual carbon formation, analyte retention, and reduced recoveries, particularly for elements associated with metalloproteins and biological complexes.

Therefore, this study aimed to develop and optimize an acid-free microwave-assisted digestion (AFMAD) method for multielement determination in complex fish matrices by ICP–MS. A multivariate optimization strategy based on central composite design (CCD) and response surface methodology (RSM) was employed to evaluate the effects of operational parameters on matrix decomposition efficiency. In addition, the analytical performance and sustainability characteristics of the proposed method were assessed in order to explore the potential of AFMAD as a greener alternative for elemental analysis of complex biological samples.

## 2 Materials and Methods

### 2.1 Materials

Trace metal standards for ICP–MS analysis for Cu (1000 ± 4 mg L^–1^), Zn (1000 mg L^–1^) and Cd (1000 mg L^–1^) were obtained from Merck (Darmstadt, Germany). Standard for Fe (1001 ± 5 mg L^–1^) was obtained from Quimlab (São Paulo, Brazil). Standards for Mn (999 ± 6 mg L^–1^) and As (1000 mg L^–1^) were obtained from Hexis Científica S/A (São Paulo, Brazil). Yttrium (Y, 1004 ± 5 mg L^–1^) and bismuth (Bi, 1000 mg L^–1^) were purchased from Fluka Analytical (Buchs, Switzerland) and employed as internal standards to evaluate variations in the introduction system. Citric acid (99.5+%), used as a standard to assess residual carbon content (RCC), was obtained from Sigma (Japan). The accuracy of the method was evaluated using certified reference material (CRM) of cod muscle (BCR-422), from the Community Bureau of Reference (BCR) (Geel, Belgium). Hydrogen peroxide (30% m/m) (Merck, Darmstadt, Germany) was exclusively used for sample preparation. All solutions and dilutions were prepared with ultrapure water obtained from Direct-Q 5 UV (Merck, Darmstadt, Germany) with resistivity of 18.2 MΩ.cm at 25 °C.

### 2.2 Sample Collection

The samples were purchased from a commercial establishment in Mariana, Minas Gerais (20°22′18.5″S, 43°24′55.6″W). Specimens of *Oreochromis niloticus* (Tilápia), *Colossoma macropomum* (Tambaqui), *Megaleporinus conirostris* (Piau), *Cichla ocellaris* (Tucunaré), and *Pseudoplatystoma corruscans* (Pintado) (Figure S1), were packed in ice and immediately transported to the laboratory for processing. The samples were thoroughly washed with deionized water to remove surface contaminants. The total length and weight of the fish were recorded (Table S1). Prior to muscle excision, titanium blades and scalpels were thoroughly decontaminated by rinsing with diluted HNO_3_ solution (1% v/v), followed by thorough rinsing with ultrapure water, to minimize potential trace-metal contamination. Muscle tissue was collected from each species and immediately transferred to clean containers. The samples were frozen at −60 °C in an ultralow-temperature freezer for 12 h and subsequently freeze-dried for 72 h using a Liotop LP510 freeze-dryer (Liobras, Brazil), with a condenser temperature of approximately −55 °C. After freeze-drying, the samples were individually packed and stored at – 20 °C until analysis.

### 2.3 Instrumentation

Microwave-assisted decomposition of the organic matter in the samples was performed using a Multiwave 5000 (Anton Paar, Graz, Austria) equipped with a rotor 20SVT50 and PTFE-TFM digestion vessels. The determination of remaining carbon was carried out through the ICP OES model iCAP 6000 series (Thermo Fisher Scientific, Bremen, Germany). Additional details regarding the operational conditions can be found in Table S2. The ICP–MS instrument used in this study was an iCAP TQ model (Thermo Fisher Scientific, Bremen, Germany) equipped with a collision/reaction cell, and with an ASX-560 autosampler (Teledyne CETAC Technologies, Omaha, NE). To ensure optimal performance, the ICP–MS was tuned daily with a solution containing Bi, Li, Co, Ce, In, and U, each at a concentration of 1 μg L^–1^. Internal standards (IS) (^209^Bi and ^89^Y, 5 μg L^–1^) were used to compensate for variations in nebulization efficiency and instrumental drift. ^89^Y was used as the internal standard for Cu, Zn and Mn, whereas ^209^Bi was used for Cd. High purity argon (99.999%) from White Martins-Praxair (São Paulo, Brazil) was used to supply the ICP–MS. Additional details regarding the operational conditions can be found in Tables S3 and S4.

### 2.4 Optimization Using Design of Experiments (DoE)

The residual carbon content was used as an indicator of the efficiency of organic matter decomposition. Lower residual carbon levels indicate a greater extent of oxidation of the organic matrix and, consequently, a lower organic load remaining in the solution after decomposition.^16^ This parameter is particularly relevant for subsequent ICP–MS analysis, since residual organic matter can affect aerosol formation and transport, alter plasma conditions, and contribute to carbon-related spectral effects.^16^ In this sense, minimizing the RCC is desirable to reduce potential matrix effects and improve the suitability of the resulting solutions for elemental analysis.

To determine the optimal conditions for AFMAD, a central composite design (CCD) was employed in conjunction with response surface methodology (RSM). Arbitrarily, the fish species Tambaqui was chosen as a representative model to perform the optimization. A 2^3^ full factorial design was implemented to evaluate the effects and interactions of the variables. The factors evaluated were: (i)­H_2_O_2_ concentration (5–30%), (ii) ramp time (6–24 min), and (iii) digestion temperature (146–213 °C). Table 1 presents the experimental design matrix, showing the actual and coded levels of the independent variables.

**Table 1:** Displays the Real and Coded Levels of the Independent Variables

variables	symbol	variable code				
		–1,68	–1	0	+1	+1,68
H_2_O_2_ concentration (%)	*x*_1_	5	10	17	25	30
ramp time (min)	*x*_2_	6	10	15	20	24
digestion temperature (°C)	*x*_3_	146	160	180	200	213

Table S5 presents the matrix for the experimental design. Analyses were carried out randomly, ensuring that uncontrollable variables were distributed throughout the experiment, avoiding bias.

Briefly, 100 mg of lyophilized fish muscle tissue was digested with 5 mL of H_2_O_2_ using microwave-assisted decomposition. The H_2_O_2_ concentration, temperature, and ramp time were established according to the experimental design (Table 1), followed by a fixed holding time of 20 min. After digestion, the solutions were filtered through 0.22 μm membrane filters and stored at 5 °C until analysis. Method blanks were prepared in parallel under identical conditions without fish tissue. The remaining carbon content in the digestates was used as the response variable to evaluate matrix decomposition efficiency. RCC was determined by ICP OES at 193.091 nm using external calibration (0–1000 mg L^–1^).

After the experiments, the data were statistically analyzed to obtain the second-order polynomial model, as described by eq 1

1 $$Y_{i}=b_{i0}+\sum_{j=1}^{n}b_{ij}X_{j}+\sum_{j=1}^{n}b_{ijj}X_{j}^{2}+\sum_{j<k=1}^{n}b_{ijk}X_{j}X_{k}$$

where *Y*_*i*_ indicates the value for a predicted response, *X*_*j*_ and *X*_*k*_ are the independent variables. *b*_*i*0_ is the constant coefficient, *b*_*ij*_ the linear coefficient, *b*_*ijj*_ the quadratic coefficient, and *b*_*ijk*_ the interaction coefficient for the specific response *Y*_*i*_. Finally, *n* represents the number of independent variables.

### 2.5 Analytical Performance

Calibration solutions with analyte concentrations ranging from 0 to 50 μg L^–1^ were prepared using aqueous standard solutions. The signal intensities (S) of each analyte were normalized by the signal intensity of the IS (^209^Bi and ^89^Y, 5 μg L^–1^), using the *S*_Analyte_/*S*_IS_ ratio to construct the discovery and quantification curves for the samples. Analytical performance parameters were determined for each analyte. The precision of the method was evaluated by calculating the relative standard deviation (%) for three consecutive measurements of each sample. The accuracy of the method was evaluated based on the agreement of the determined analyte concentrations (*C*_det_) (eq 2) with the certified concentrations (*C*_cert_) obtained for the certified reference material (CRM) of cod muscle (BCR-422), provided by the Community Bureau of Reference (BCR, Geel, Belgium). The CRM was analyzed using the same analytical procedure applied to the samples, and the determined concentrations were compared with the corresponding certified values. The agreement between the experimental and certified concentrations demonstrates the accuracy of the proposed analytical procedure and provides an appropriate quality assurance measure.

2 $$Recovery\left(\%\right)=\frac{C_{\det}}{C_{cert}}\times 100$$

The LOD was estimated according to the recommendations of the International Union of Pure and Applied Chemistry (eq 3), as three times the standard deviation of the blank signal (Sb, *n* = 10) divided by the slope of the calibration curve equation (*m*). Similarly, the LOQ (Eq. 4) was defined as ten times Sb (*n* = 10) divided by *m* (slope).

3 $$LOD=\frac{3\times Sb}{m}$$

4 $$LOQ=\frac{10\times Sb}{m}$$

### 2.6 Statistical Analysis

The experiments were conducted in a randomized sequence to minimize systematic errors. Subsequent analyses and the validation process were performed in triplicate. Statistical comparisons between the mean values obtained in different tests were performed with a 95% confidence level. Student’s *t*-test or analysis of variance (ANOVA) were used to statistically assess possible differences. Data processing was performed using STATISTICA 7 software (StatSoft, Inc., Tulsa, OK, USA).

## 3 Results and Discussion

### 3.1 Multivariate Optimization of AFMAD

A full two-level factorial design (2^3^) followed by a central composite design (CCD) with response surface methodology (RSM) was used to optimize the decomposition conditions, using the remaining carbon content as the response variable. Carbon quantification showed excellent linearity over the evaluated concentration range (*R* ^2^ = 0.999). The experimental design is presented in Table S6, and the corresponding response surface and contour plots are shown in Figure 1.

**Figure 1:**
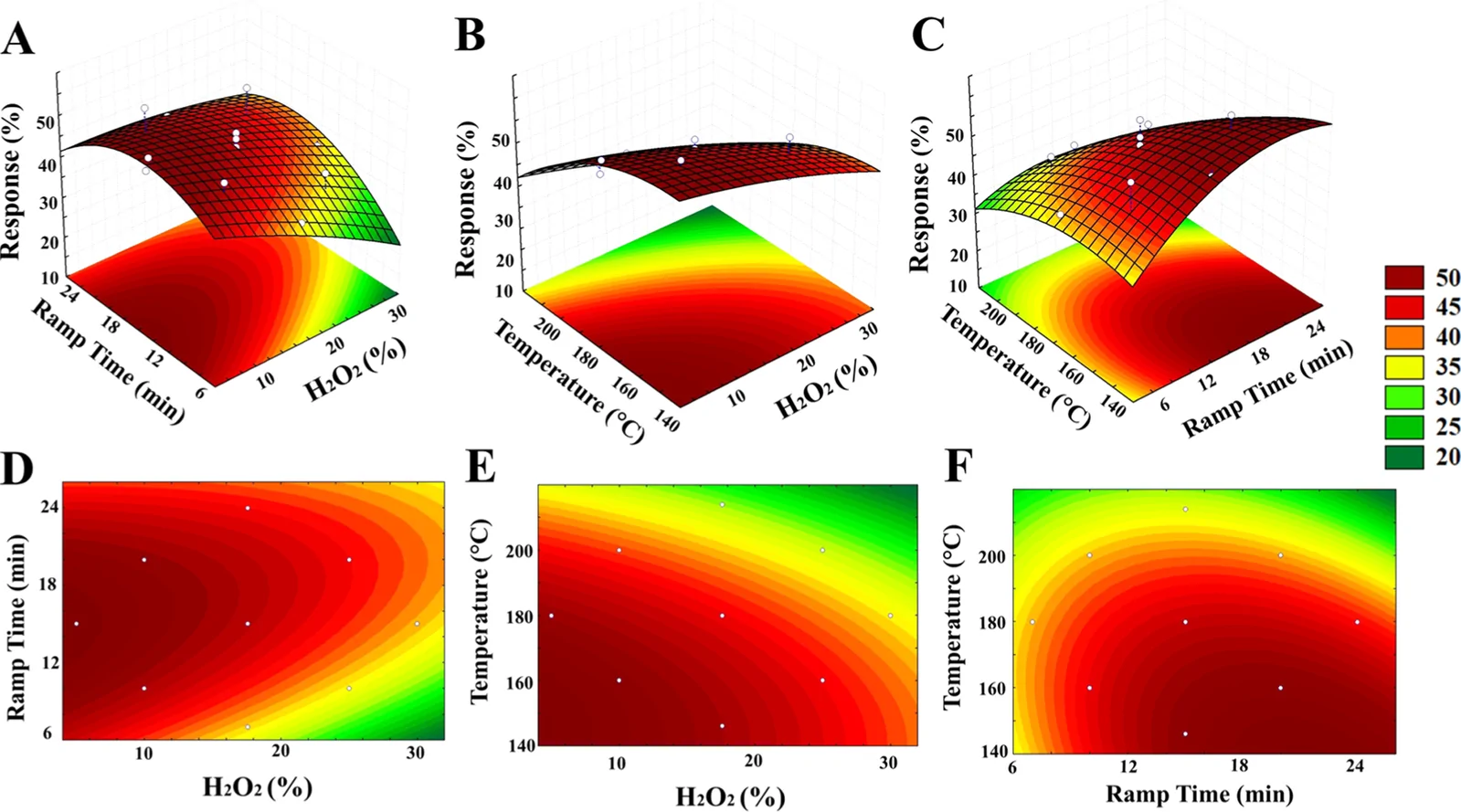
Response surface plots (A–C) and corresponding contour plots (D–F) illustrating the interactive effects of hydrogen peroxide concentration (H_2_O_2_% v/v), ramp time (min), and temperature (°C) on the remaining carbon content (response, %). Color gradients represent the percentage of residual carbon, from low (green) to high (reddish-brown) values.

The relationship between the response (residual carbon content) and the independent variables was established by the following second-order polynomial equation (eq 5)­

5 $$Y=-73.5579-0.5725x_{1}+5.2912x_{2}+1.2174x_{3}-0.0075x_{1}^{2}-0.0846x_{2}^{2}-0.0034x_{3}^{2}+0.0325x_{1}x_{2}-0.0010x_{1}x_{3}-0.0162x_{2}x_{3}$$

where *x*_1_ represents the H_2_O_2_ concentration (%), *x*_2_ the ramp time (min), and *x*_3_ the hold temperature (°C). ANOVA indicated that all three individual variables have a statistically significant effect on the response variable with a 95% confidence level (Table 2).

**Table 2:** Parameters Adjusted from an ANOVA Regression Using the Results of a Central Composite Design for Optimization of AFMAD Decomposition

factor^a^	sum quadratic (SS)	degrees of freedom	*F*_calc_^b^	*P*
*x*_1_	211.23	1	155.78	0.0011
*x*_2_	54.54	1	40.22	0.0080
*x*_3_	353.05	1	260.37	0.00052
interaction *x*_1_ *vs x*_2_	11.90	1	8.78	0.060
interaction *x*_1_ *vs x*_3_	0.18	1	0.13	0.74
interaction *x*_2_ *vs x*_3_	21.09	1	15.55	0.029
lack of fit	10.93	5	1.61	0.37
pure error	4.07	3		
residue	726.47	17		
R^2^	0.98			
R adjusted	0.96			

H_2_O_2_ concentration and temperature significantly affected the RCC, with higher values improving matrix decomposition and reducing the residual carbon content. In contrast, longer ramp times increased the RCC, indicating that shorter ramp times are more suitable for efficient digestion. Higher H_2_O_2_ concentrations and temperatures may favor oxidative matrix decomposition through reactive oxygen species, which are highly reactive toward organic components such as proteins and lipids.^17,18^ The Pareto chart (Figure 2A) corroborates these findings by highlighting the relative contribution of the evaluated factors.

**Figure 2:**
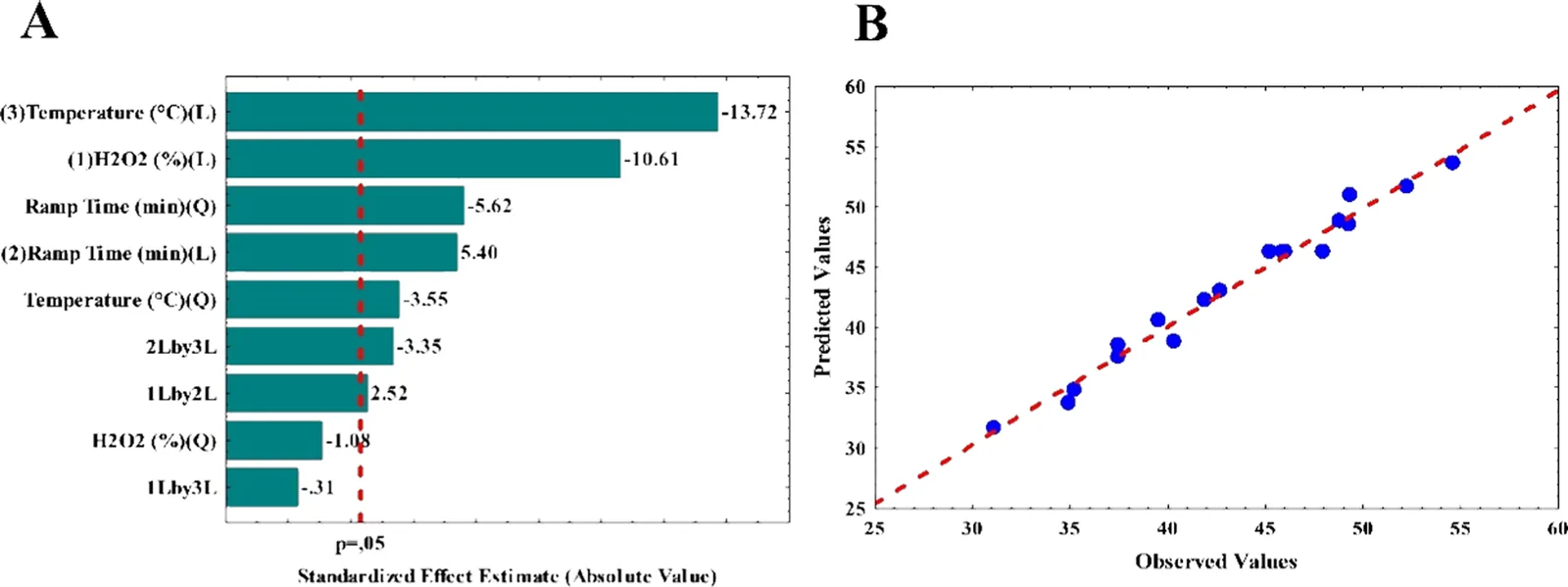
(A) Pareto chart of standardized effects showing the relative significance of individual variables and their interactions affecting the response variable (residual carbon content). Statistically significant variables exceed the threshold of *p* = 0.05. (B) Predicted versus observed residual carbon content values, according to an ANOVA regression with a 95% confidence level, demonstrating the good fit of the empirical model used. Q and L represent quadratic and linear effects, respectively. 1, 2, and 3 correspond to H_2_O_2_ concentration, ramp time, and hold temperature, respectively. 1L by 2L, 2L by 3L, and 1L by 3L represent interactions between individual variables with a linear effect.

The predicted versus observed plot (Figure 2B) demonstrated good agreement between experimental and predicted RCC values, confirming the adequacy of the quadratic model (*R* ^2^ = 0.98, no significant lack of fit). Based on the response surface analysis, the optimal AFMAD conditions were established as 30% (v/v)­H_2_O_2_, an 8 min ramp time, and a hold temperature of 240 °C. The selected hold temperature of 240 °C was obtained by extrapolation of the fitted mathematical model, which indicated a continued decrease in RCC at higher temperatures. Under these conditions, the model predicted an RCC below 20%, indicating efficient oxidation of the organic matrix using H_2_O_2_ in the absence of HNO_3_. Representative images of the final visual aspect samples are provided in Figure S2.

### 3.2 Evaluation of Figures of Merit

The analytical performance of the proposed AFMAD method was evaluated using standard figures of merit, including LOD, LOQ, linearity, linear working range, and sensitivity (Table S7). Quantification was performed by external calibration using standard solutions prepared from multielement standards. The linear analytical curves for all analytes exhibited excellent linearity, with coefficients of determination (R^2^) higher than 0.99. The obtained LOD and LOQ values were suitable for trace-level elemental determination in fish matrices.

The optimized AFMAD conditions were applied to the analysis of CRM from cod muscle (BCR-422). The results are presented in Table 3.

**Table 3:** Total Concentrations (mg kg^–1^) of Target Metals (Mean Value ±Standard Deviation, *n* = 3) in Certified Reference Material From Cod Muscle (BCR CRM-422) Using ICP–MS^a^

		total content			
analyte	certified (mg kg^–1^)	determined (mg kg^–1^)	recovery (%)	RSD (%)	*t*_calc_ ^b^
Fe	5.5 ± 0.3	4.2 ± 0.2	76	4.50	11.86
Cu	1.05 ± 0.07	1.03 ± 0.07	98	6.80	0.42
Zn	19.6 ± 0.5	19.8 ± 1.7	101	8.59	0.19
Mn	0.543 ± 0.028	0.43 ± 0.02	80	4.65	8.60
As	21.1 ± 0.5	20.0 ± 1.2	95	5.81	1.72
Cd	0.017 ± 0.002	0.015 ± 0.001	88	6.67	3.53

a Student’s *t*-test (*T*_calc_) values, calculated to evaluate the differences between certified and experimentally determined values, are also presented.

b Critical *t*-value: 4.303.

Recoveries for these elements ranged from 76% to 101%. For most analytes (Cu, Zn, As, and Cd), no statistically significant differences were observed between the determined and certified values, since the calculated *t*-values were lower than the critical value (*t*_crit_ = 4.303, α = 0.05). For Fe and Mn, the *t*_calc_ values (11.856 and 8.597, respectively) were higher than the critical value, indicating significant difference. However, despite these differences, the obtained recoveries for these elements still fall within the acceptable validation criteria established by international guidelines for trace elemental analysis in complex matrices.^19^ Additionally, Fe is frequently associated with metalloproteins and insoluble biological complexes, which may require stronger oxidative and hydrolytic conditions for complete release.^20^ In conventional acid digestion, nitric acid contributes not only to oxidation but also to the disruption of coordination environments and stabilization of metallic species in solution. In the absence of HNO_3_, partial retention of Fe in refractory organic residues may occur, which helps explain the lower recovery observed for this analyte.

The precision of the method was evaluated through the relative standard deviation (RSD). For all analytes, RSD values were below 10%, demonstrating excellent repeatability for trace analysis in biological matrices. These results demonstrate the applicability of the proposed AFMAD method for multielement determination in complex fish matrices. Overall, the method is suitable for multielement screening and routine monitoring, although limitations remain for elements associated with metalloproteins and other biomolecular species, such as Fe and Mn, which may require more aggressive digestion conditions for complete recovery. The AFMAD method demonstrated favorable analytical performance while avoiding the use of concentrated mineral acids. The satisfactory analytical performance indicates its potential for routine multielement determination in complex solid samples, such as fish, while adhering to the principles of green chemistry.

### 3.3 Application to Real Samples

After validating the analytical performance, the applicability of the proposed AFMAD method was evaluated through the analysis of real samples. Muscle tissues from commercial fish of different species were selected as representative complex matrices to demonstrate the robustness and precision of the method. Table 4 summarizes the results obtained for the different species using AFMAD method.

**Table 4:** Total Concentrations (mg kg^–1^) of Target Metals (Mean Value ±Standard Deviation, *n* = 3) Determined in Real Samples Using AFMAD and ICP–MS

	total content (mg kg^–1^)					
sample	Fe	Cu	Zn	Mn	As	Cd
Colossoma macropomum (Tambaqui)	6.39 ± 0.03	0.48 ± 0.01	8.82 ± 0.06	0.42 ± 0.06	<0.001	<0.002
Oreochromis niloticus (Tilápia)	6.81 ± 0.7	0.60 ± 0.006	10.70 ± 0.23	0.26 ± 0.008	<0.001	<0.002
Megaleporinus conirostris (Piau)	9.57 ± 0.03	0.88 ± 0.1	16.02 ± 0.6	0.63 ± 0.02	<0.001	0.003 ± 0.0008
Pseudoplatystoma corruscans (Pintado)	5.84 ± 0.1	0.50 ± 0.03	13.27 ± 1.09	0.19 ± 0.01	<0.001	<0.002
Cichla ocellaris (Tucunaré)	3.54 ± 0.1	0.58 ± 0.01	13.88 ± 0.1	0.39 ± 0.02	0.07 ± 0.008	<0.002

The results of the multielement analysis revealed a predominance of Fe and Zn for all species analyzed, establishing a general abundance hierarchy of Zn > Fe > Cu > Mn. It is important to note that this order is presented to illustrate the elemental distribution observed in the analyzed samples. *Megaleporinus conirostris* stood out for presenting the highest concentrations of Fe (9.57 mg kg^–1^), Cu (0.88 mg kg^–1^), Zn (16.02 mg kg^–1^), and Mn (0.63 mg kg^–1^), also being the only species with detectable Cd (0.003 mg kg^–1^). On the other hand, As was exclusively quantified in *C. ocellaris* (0.07 mg kg^–1^).^21^ *C. macropomum* exhibited the lowest overall concentrations, with the lowest contents of Cu (0.48 mg kg^–1^) and Zn (8.82 mg kg^–1^), and levels of As, and Cd below the LOD of the method.

Differences in feeding ecology among the analyzed species may partly contribute to variations in elemental accumulation. Fish can be exposed to metals through multiple pathways, including water, sediments, and diet, while feeding strategy, habitat, trophic position, and species-specific physiological characteristics can influence their accumulation.^22^ Dietary exposure may represent an important route of metal uptake, particularly for species at higher trophic levels.^22^ Carnivorous species, such as *C. ocellaris* and *P. corruscans*, occupy higher trophic positions than omnivorous and detritivorous species, which may increase their susceptibility to dietary exposure and trophic transfer of some elements.^23^ In contrast, *C. macropomum* has a predominantly frugivorous/omnivorous feeding strategy, with fruits, seeds, and zooplankton representing important dietary components.^24^ Such differences in food sources and trophic position can influence exposure pathways and elemental accumulation.

To evaluate the safety for human consumption, the metal contents were compared with the Maximum Tolerated Limits (MTL) established by Agência Nacional de Vigilância Sanitária (ANVISA, Normative Instruction No. 160/2022) of Brazil^25^ and the guidelines of the Food and Agriculture Organization of the United Nations (FAO). The concentrations of As and Cd across all analyzed fish species were significantly lower than the established MTLs. The highest As concentration, found in *C. ocellaris* (0.07 mg kg^–1^), represents only 7% of the limit permitted by ANVISA (1.0 mg kg^–1^). The occurrence of As exclusively in *C. ocellaris* may be associated with its predatory feeding behavior and higher trophic position, which can favor biomagnification processes in aquatic ecosystems.^21^ Similarly, the Cd level detected in *Megaleporinus conirostris* (0.003 mg kg^–1^) was well below the threshold for fish muscle (0.05 mg kg^–1^). According to FAO guidelines (Codex Alimentarius), there are no maximum levels (MLs) established for Fe, Cu, Zn and Mn in fish tissues, as the former four are classified as essential nutrients. Specifically, the maximum levels (MLs) for contaminants followed the General Standard for Contaminants and Toxins in Food and Feed (CXS 193–1995).^26^ The absence of regulatory limits for these elements, coupled with the low concentrations found in this study, reinforces the safety of the analyzed species regarding their mineral contribution and the absence of acute toxicity for these analytes.

These trace elements, even at low concentrations, pose severe ecological and human health risks due to their potential to bioaccumulate throughout the trophic chain, ultimately affecting top predators and human consumers.^27^ The AFMAD method demonstrated the required analytical sensitivity to track these trace levels before they reach critical thresholds, serving as a highly effective preventive monitoring tool.

## 4 Sustainable Analytical Chemistry Metrics

To quantitatively highlight the environmental and operational advantages of the proposed approach, the analytical greenness of the sample preparation step was evaluated using the AGREEprep tool (analytical GREEnness metric for sample preparation) developed by Wojnowski et al.^28^ (obtained from https://agreeprep.anvil.app/). In order to position the proposed AFMAD strategy within the current state of the art, other MAD methods were also assessed using the using the AGREEprep metric. In this context, the proposed H_2_O_2_-based decomposition procedure was compared with seven acid–based digestion methods reported in the literature.^29–32^ For studies in which the number of samples processed simultaneously was not explicitly described, the nominal rotor capacity of the microwave digestion system was considered to estimate sample throughput and energy consumption per sample. The AGREEprep pictograms obtained for the evaluated methods are presented in Figure 3, while additional information regarding the literature procedures is provided in Table S8.

**Figure 3:**
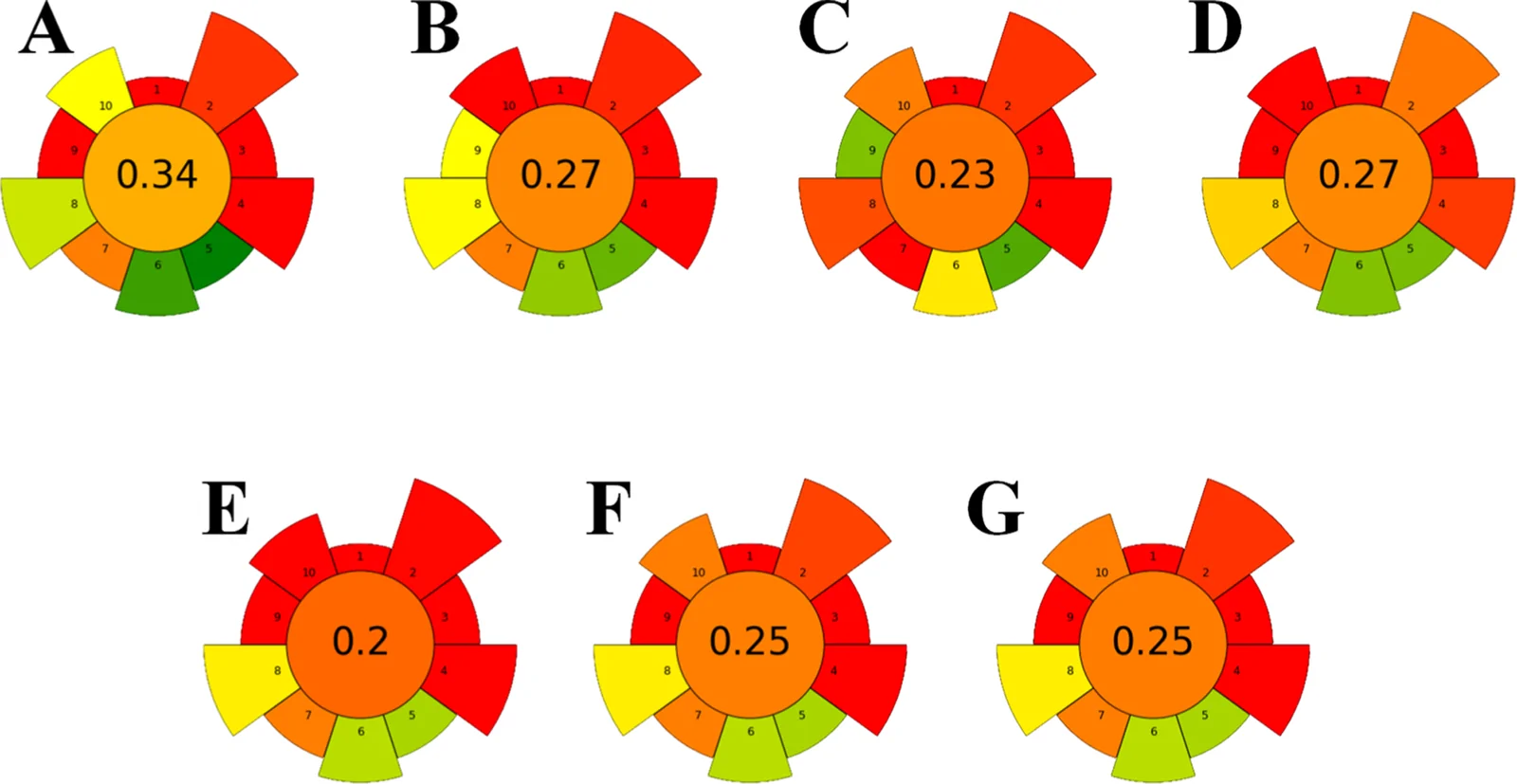
AGREEprep scores obtained for different sample preparation strategies applied to complex biological matrices. (A) Proposed AFMAD method using H_2_O_2_ for fish tissue decomposition (this work), (B) microwave-assisted digestion using HNO_3_ + HCl for fish tissue samples,^29^ (C) HNO_3_ digestion for tuna and squid samples,^30^ (D) HNO_3_ + H_2_O_2_ digestion for bivalve samples,^31^ and fish tissue digestion using (E) HNO_3_ + H_2_O_2_, (F) HNO_3_ + H_2_O, and (G) HNO_3_ procedures.^32^

Methods based on concentrated HNO_3_ generally exhibited lower greenness scores due to the use of hazardous reagents. In contrast, procedures employing reduced acid volumes or alternative oxidizing systems generally achieved higher greenness scores. The AFMAD method proposed in this study showed one of the highest scores among the assessed strategies, mainly due to the exclusive use of H_2_O_2_ as oxidizing reagent during sample preparation.

Among the parameters evaluated, AFMAD showed good performance particularly regarding sample size economy (criterion 5) and the sample throughput criterion (criterion 6) of the AGREEprep assessment. The elimination of concentrated HNO_3_ also represents a positive environmental and operational advantage by reducing hazards associated with handling highly corrosive reagents, thereby enhancing laboratory safety.^13^

The use of H_2_O_2_ reduces the production of hazardous waste and byproducts, since its main decomposition products are water and oxygen. Beyond reducing hazardous reagent consumption, the elimination of concentrated mineral acids also minimizes maintenance associated with corrosion damage, improves laboratory safety, and reduces risks associated with large-scale environmental monitoring campaigns.

It is important to highlight that the comparison with dilute-acid digestion procedures should consequently be interpreted with caution, since differences in filtration procedures can contribute to differences in the apparent RCC values. Nevertheless, filtration was intentionally incorporated as a practical postdigestion step to obtain particle-free solutions suitable for ICP–MS analysis and represents an operational consideration of the proposed method rather than a modification of the microwave digestion chemistry itself.

## 5 Conclusions

This study demonstrated the feasibility of applying acid-free microwave-assisted digestion (AFMAD) for multielement determination in complex fish tissues by ICP–MS. The proposed strategy, based exclusively on H_2_O_2_ as oxidizing reagent, provided satisfactory analytical performance while eliminating the use of concentrated mineral acids, contributing to safer and more sustainable sample preparation procedures.

The multivariate optimization based on central composite design and response surface methodology allowed the identification of operational conditions capable of promoting efficient matrix decomposition under fully acid-free conditions. The proposed method showed suitable linearity, sensitivity, precision, and satisfactory recoveries for most evaluated analytes, demonstrating its applicability for trace elemental analysis in complex biological matrices. Although lower recoveries were observed for Fe and Mn, likely due to their association with biological complexes and incomplete release under mild oxidative conditions, the method still demonstrated promising performance for multielement screening and monitoring purposes.

Application of the AFMAD procedure to fish samples collected in the Mariana region confirmed its potential for environmental and food safety assessment, with toxic element concentrations remaining below the limits established by Brazilian regulations. In addition, the AGREEprep evaluation highlighted the favorable sustainability characteristics of the proposed approach compared with conventional acid–based digestion procedures.

Overall, the proposed AFMAD method represents a promising greener alternative for elemental analysis of complex biological samples, particularly in applications requiring reduced hazardous reagent consumption, lower secondary waste generation, and improved operational safety.

## Supplementary Material



## Notes

Fish species commercially obtained in Mariana, Minas Gerais (Figure S1); body weight and total length (Table S1); ICP-OES and ICP–MS operating conditions (Tables S2–S3); monitored *m*/*z* ratios and acquisition modes (Table S4); factorial design matrix (Table S5); optimization results (Table S6); representative digests (Figure S2); figures of merit (Table S7); and microwave-assisted decomposition procedures for AGREEprep evaluation (Table S8) (PDF)

## References

[1] Pacheco F. A. L., do Valle Junior R. F., de Melo Silva M. M. A. P., Pissarra T. C. T., Carvalho de Melo M., Valera C. A., Sanches Fernandes L.
F. (2022). Prognosis of metal concentrations
in sediments and water of Paraopeba River following the collapse of
B1 tailings dam in Brumadinho (Minas Gerais, Brazil). Sci. Total Environ..

[2] Rezende V. L. (2016). A mineração
em Minas Gerais: uma análise de sua expansão e os impactos
ambientais e sociais causados por décadas de exploração. Rev. Sociedade Natureza.

[3] Enyoh C. E., Verla A. W., Qingyue W., Ohiagu F. O., Chowdhury A. H., Enyoh E. C., Chowdhury T., Verla E. N., Chinwendu U. P. (2020). An overview
of emerging pollutants in air: Method of analysis and potential public
health concern from human environmental exposure. Trends Environ. Anal. Chem..

[4] Inobeme A., Mathew J. T., Jatto E., Inobeme J., Adetunji C. O., Muniratu M., Onyeachu B. I., Adekoya M. A., Ajai A. I., Mann A., Olori E., Akhor S. O., Eziukwu C. A., Kelani T., Omali P. I. (2023). Recent
advances in instrumental techniques
for heavy metal quantification. Environ. Monit.
Assess..

[5] Pereira, M. V. d. S. , J. R., de Jesus , Metallospecies and their biological functions in Alzheimer’s disease, in: 2026. 193, 217, DOI: 10.1016/bs.irn.2026.02.005. 42442912

[6] Rahman Z., Singh V. P. (2019). The relative impact
of toxic heavy metals (THMs) (arsenic
(As), cadmium (Cd), chromium (Cr)­(VI), mercury (Hg), and lead (Pb))
on the total environment: an overview. Environ.
Monit. Assess..

[7] Zhou X., Wu Z., Chen B., Zhou Z., Liang Y., He M., Hu B. (2024). Quantification
of trace heavy metals in environmental water, soil
and atmospheric particulates with their bioaccessibility analysis. Talanta.

[8] Liu J., Zheng L., Wei X., Wang B., Chen H., Chen M., Wang M., Feng W., Wang J. (2022). Quantitative
imaging of trace elements in brain sections of Alzheimer’s
disease mice with laser ablation inductively coupled plasma-mass spectrometry. Microchem. J..

[9] Arruda M. A. Z., de Jesus J. R., Blindauer C. A., Stewart A. J. (2022). Speciomics as a
concept involving chemical speciation and omics. J. Proteomics.

[10] Bağda E., Altundağ H., Keskin C. S. (2022). Deep Eutectic Solvent-based Extraction
of As, Mn, Pb, Cr, and Ni from Spleen, Kidney, and Brain Samples. J. Anal. Chem..

[11] Grochowski C., Blicharska E., Krukow P., Jonak K., Maciejewski M., Szczepanek D., Jonak K., Flieger J., Maciejewski R. (2019). Analysis of
Trace Elements in Human Brain: Its Aim, Methods, and Concentration
Levels. Front. Chem..

[12] Fleischer, H. ; Horn, S. ; Bruckhaus, J. J. ; Klinder, A. ; Lutter, C. ; Mittelmeier, W. ; Büttner, A. ; Thurow, K. Multi-element and Multi-organ Heavy Metal Measurements Using ICP-MS - A Contribution to Reference Value Generation in Human Tissues. In 2025 IEEE International Instrumentation and Measurement Technology Conference (I2MTC); IEEE, 2025. 10.1109/I2MTC62753.2025.11079088.

[13] de
Jesus J. R., Yang K. X., Donati G. L. (2025). Green sample preparation
method based on acid-free sonochemical extraction for trace element
determination in biological samples. Microchem.
J..

[14] da
Silva A. B. S., da Silva Leal K. N., Arruda M. A. Z. (2025). An acidless microwave-assisted
wet digestion of biological samples as a greener alternative: applications
from COVID-19 monitoring to plant nanobiotechnology. Anal. Bioanal. Chem..

[15] Arbova D. L., Tolmachev S. Y., Brockman J. D. (2022). Quadrupole and multi-collector ICP-MS
analysis of 226Ra in brain from a radium dial painter. J. Anal. At. Spectrom..

[16] Muller E.
I., Souza J. P., Muller C. C., Muller A. L. H., Mello P. A., Bizzi C. A. (2016). Microwave-assisted
wet digestion with H2O2 at high
temperature and pressure using single reaction chamber for elemental
determination in milk powder by ICP-OES and ICP-MS. Talanta.

[17] Pooja G., Shweta S., Patel P. (2025). Oxidative
stress and free radicals
in disease pathogenesis: a review. Discover
Medicine.

[18] Cao X., Xu M., Feng T., Li R., Song Y., Meng N., Fan X., Zeng J., Xu J. (2024). A comparative lipid profile of four
fish species: From muscle to industrial by-products based on RPLC–Q-TOF-MS/MS. Food Res. Int..

[19] AOAC International Guidelines for Standard Method Performance Requirements, 22nd ed.; Association of Official Analytical Chemists: Rockville, 2023.

[20] Pereira, M. V. d. S. , J. R., de Jesus , Metallospecies and their biological functions in Alzheimer’s disease, in: 2026. 193, 217. 10.1016/bs.irn.2026.02.005. 42442912

[21] Guedes G. H. S., Franco A. C. S., dos Santos L. N., dos Santos A. F. G. N., Araújo F. G. (2024). Cannibalism facilitates and sustains
the invasion of top-predator fish (Cichlidae: Cichla) in human-modified
ecosystems. Freshw. Biol..

[22] Oros A. (2025). Bioaccumulation
and Trophic Transfer of Heavy Metals in Marine Fish: Ecological and
Ecosystem-Level Impacts. J. Xenobiot..

[23] Rodrigues B. L., Canto A. C. V. d. C. S., Costa M. P. d., Silva F. A. d., Mársico E. T., Conte-Junior C. A. (2017). Fatty acid profiles of five farmed Brazilian freshwater
fish species from different families. PLoS One.

[24] SILVA J. A. M. d., Pereira-Filho M., Oliveira-Pereira M. I. d. (2000). Seasonal
variation of nutrients and energy in tambaqui’s (Colossoma
Macropomum Cuvier, 1818) natural food. Rev.
Bras. Biol..

[25] Brazil ., Normative Instruction No. 160/2022. It establishes the maximum tolerated limits (MTL) of contaminants in food. https://www.gov.br/agricultura/pt-br/assuntos/inspecao/produtos-animal/plano-de-nacional-de-controle-de-residuos-e-contaminantes/instrucao-normativa-anvisa-2022_160-1.pdf/@@download/file (accessed March 16, 2026).

[26] Codex Alimentarius Commission , General Standard for Contaminants and Toxins in Food and Feed (CXS 193–1995), 1995. https://www.fao.org/fao-who-codexalimentarius/sh-proxy/en/?lnk=1&url=https%253A%252F%252Fworkspace.fao.org%252Fsites%252Fcodex%252Fstandards%252FCXS%2B193-1995%252FCXS_193e.pdf (accessed March 16, 2026).

[27] Naz S., Habib S. S., Arshad M., Majeed S., Acar U. ¨., Kesbiç O. S., Mohany M., Aragona F., Fazio F. (2025). Human health
risk of heavy metal biomagnification: Trophic transfer
patterns in aquatic ecosystems. J. Trace Elem.
Med. Biol..

[28] Wojnowski W., Tobiszewski M., Pena-Pereira F., Psillakis E. (2022). AGREEprep
– Analytical greenness metric for sample preparation. TrAC, Trends Anal. Chem..

[29] Pothuraju N., Pogula H. K., Jagdale R., Vadla U. K., Gajbhiye R. L., Parihar V. K., Velayutham R., Peraman R. (2025). Impact of microwave-assisted
acid extraction (MW-AAE) methods on simultaneous ICP-MS analysis of
multi-elements in edibles besides associated greenness and human health
risk assessment. Environ. Monit. Assess..

[30] Singh P. P., Gupta S. M. (2024). Comparison between Microwave Digestion
and Conventional
Open Digestion Method for Heavy Metals determination. Ecol., Environ. Conserv..

[31] Šteković M., Pucko I., Faraguna F., Stankov V., Verbanac D. (2026). Development
of a Microwave-Assisted Digestion Procedure for Microplastics Extraction
from Different Food Matrices with Subsequent Analysis Using Raman
Microspectroscopy. Microplastics.

[32] Sirhan A., Jarrar Q., Ayoub R., Abudayeh Z., Alsaloumi L., Hantash T., Mostafa A., Ghanem R., Hassouneh L., Abdulra’uf L.
B. (2026). Determination and Risk
Evaluation of Heavy Metals in
Fish Samples Using AAS After Microwave-Assisted Digestion. Current Research in Nutrition and Food Science Journal.

