# Supplementary material for: A Greener Acid-free Microwave-Assisted Digestion Method for Multielement Analysis of Fish Tissues by ICP–MS
Source: ACS Omega. 2026 Oct 19;11(43):67308–16. doi: 10.1021/acsomega.6c08049 (PMC13737882; doi:10.1021/acsomega.6c08049)
Supplement: Supplementary file 1 [file ao6c08049_si_001.pdf]

SUPPLEMENTARY INFORMATION

A greener acid-free microwave-assisted digestion method for multielement analysis of fish tissues by ICP-MS

Marcos V. S. Pereira[a,c], Elisânia K. B. Fonseca[b,c], Jemmyson R. de Jesus[a,c*], Marco A. Z. Arruda[b,c**]

[a]Research Laboratory in bionanomaterials, LPbio, Department of Chemistry, Federal University of Viçosa, 36570-900 Viçosa, Minas Gerais, Brazil

[b]Group of Spectrometry, Sample Preparation and Mechanization, GEPAM, Department of Analytical Chemistry, Institute of Chemistry, University of Campinas - UNICAMP, Campinas, 13083-970, SP, Brazil

[c]National Institute of Science and Technology for Bioanalytics-Lauro Kubota, INCTBio-LK, 13083970 Campinas, São Paulo, Brazil

*Prof. Jemmyson R. de Jesus (jemmyson.jesus@ufv.br)

** Prof. Marco A. Z. Arruda (zezzi@unicamp.br)

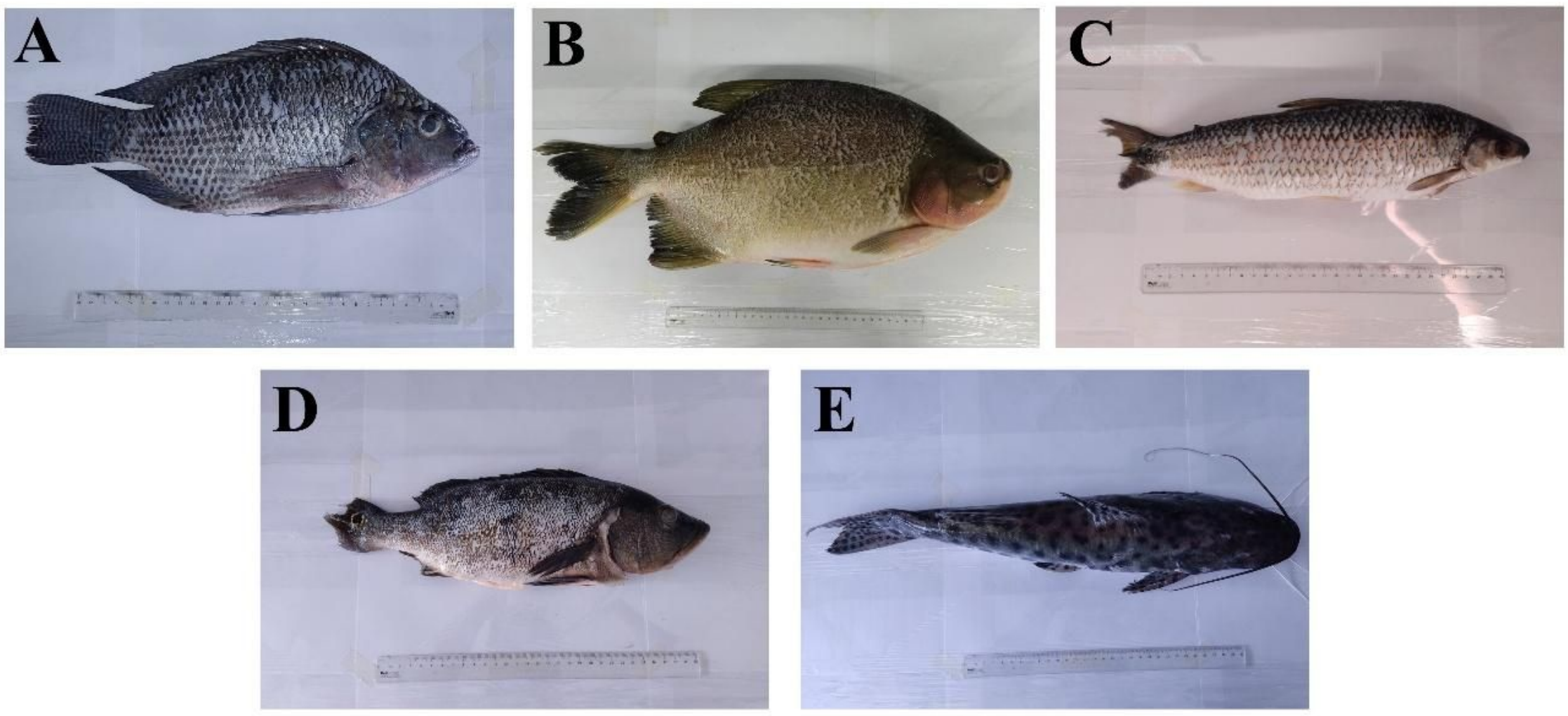

Figure S1. Fish species collected in Mariana, Minas Gerais, used in the study. (A) *Oreochromis niloticus* (Tilapia), (B) *Colossoma macropomum* (Tambaqui), (C) *Leporinus conirostris* (Piau), (D) *Cichla ocellaris* (Tucunaré) and (E) *Pseudoplatystoma corruscans* (Pintado).

Table S1. Body weight and total length of fish species commercially obtained in Mariana, Minas Gerais.

	Weight (kg)	Width (cm)	Length (cm)
Oreochromis niloticus (Tilápia)	0.87	12.86	28.84
Colossoma macropomum (Tambaqui)	3.74	22.41	46.99
Leporinus conirostris (Piau)	0.60	8.38	33.21
Cichla ocellaris (Tucunaré)	0.88	10.05	31.94
Pseudoplatystoma corruscans (Pintado)	1.18	9.23	44.67

Table S2. ICP OES instrumental operating conditions used for carbon determination.

Instrument parameter	Operating conditions
**Plasma**	
Radio frequency (RF) applied power (kW)	1.15
Plasma viewing mode	Axial
Nebulization gas flow rate (L min^{-1})	0.70
Plasma gas flow rate (L min^{-1})	15.0
Auxiliary gas flow rate (L min^{-1})	0.5
**Other analytical parameters**	
Pump stabilization time (s)	15
Sample flush time (s)	30
Analysis pump rate (rpm)	20
Number of instrument replicates	3
Analyte (emission wavelength in nm)[a]	C (193.091)^{I}

[a]I represent atomic emission line

Table S3. ICP MS instrumental operating conditions

Instrument parameter	Operating conditions
**Plasma**	
Radio frequency (RF) applied power (kW)	1.55
Sampling depth (mm)	5.5
Plasma viewing mode	axial
Nebulization gas flow rate (L min^{-1})	1.02
Plasma gas flow rate (L min^{-1})	14.0
Auxiliary gas flow rate (L min^{-1})	0.80
**Other analytical parameters**	
Number of instrumental replicates	3
Number of sweeps	10
Uptake delay (s)	50
Peristaltic pump speed (rpm)	30
Sample flow rate (mL min^{-1})	1.4
He gas flow (KED) (mL min^{-1})	4.20
O_2 gas flow (mL min^{-1})	0.28
H_2 gas flow (mL min^{-1})	10.5
Introduction system	quartz cyclonic spray chamber (2.5 ºC) and borosilicate glass concentric nebulizer (Micromist model)
Isotopes	^{55}Mn, ^{56}Fe, ^{63}Cu, ^{64}Zn, ^{89}Y, ^{114}Cd, ^{75}As, and ^{209}Bi

The reaction/collision cell (CRC) was pressurized with helium (He) and operated in the kinetic energy discrimination (KED), oxygen (O_2) and hydrogen (H_2) to reduce the spectral interferences. He-KED was used for ^{55}Mn, ^{63}Cu, ^{64}Zn and ^{114}Cd. The analysis of ^{56}Fe was performed using H_2 as reaction gas. The analysis of ^{75}As was performed using O_2 as reaction gas, in the mass-shift mode (*m/z* 91), see Table S4.

Table S4. Monitored mass charge ratio (m/z) and acquisition mode in the ICP-MS

	1st Quadrupole	Collision/Reaction Cell	2nd Quadrupole
Mn	NDA*	KED (He)	^{55}Mn
Fe	^{56}Fe	H_2	^{56}Fe
Cu	NDA*	KED (He)	^{63}Cu
Zn	NDA*	KED (He)	^{64}Zn
As	^{75}As	O_2	$^{75}As^{16}O$
Cd	NDA*	KED (He)	^{114}Cd

*NDA – No m/z filtered

Table S5. Complete matrix of the factorial design (2^3) with central and axial points, generated by the STATISTICA software.

Run	H_2O_2 concentration (%)	Ramp time (min)	Digestion temperature (°C)
5	1.00	-1.00	-1.00
8	1.00	1.00	1.00
12	0.00	1.68	0.00
14	0.00	0.00	1.68
16 (C)	0.00	0.00	0.00
3	-1.00	1.00	-1.00
7	1.00	1.00	-1.00
10	1.68	0.00	0.00
13	0.00	0.00	-1.68
4	-1.00	1.00	1.00
2	-1.00	-1.00	1.00
1	-1.00	-1.00	-1.00
15 (C)	0.00	0.00	0.00
9	-1.68	0.00	0.00
17 (C)	0.00	0.00	0.00
6	1.00	-1.00	1.00
11	0.00	-1.68	0.00
18 (C)	0.00	0.00	0.00

Table S6. Results of multifactorial experiments used to optimize the acid-free microwave-assisted decomposition method.

Run	H_2O_2 concentration (%)	Ramp time (min)	Digestion temperature (°C)	Response (%)
5	25.00	10.00	160.00	40.3
8	25.00	20.00	200.00	35.2
12	17.60	23.41	180.00	42.7
14	17.60	15.00	213.64	34.9
16 (C)	17.60	15.00	180.00	45.2
3	10.10	20.00	160.00	54.6
7	25.00	20.00	160.00	49.3
10	30.13	15.00	180.00	37.5
13	17.60	15.00	146.36	49.3
4	10.10	20.00	200.00	39.5
2	10.10	10.00	200.00	41.9
1	10.10	10.00	160.00	48.8
15 (C)	17.60	15.00	180.00	45.9
9	5.07	15.00	180.00	52.2
17 (C)	17.60	15.00	180.00	47.9
6	25.00	10.00	200.00	31.1
11	17.60	6.59	180.00	37.5
18 (C)	17.60	15.00	180.00	46.0

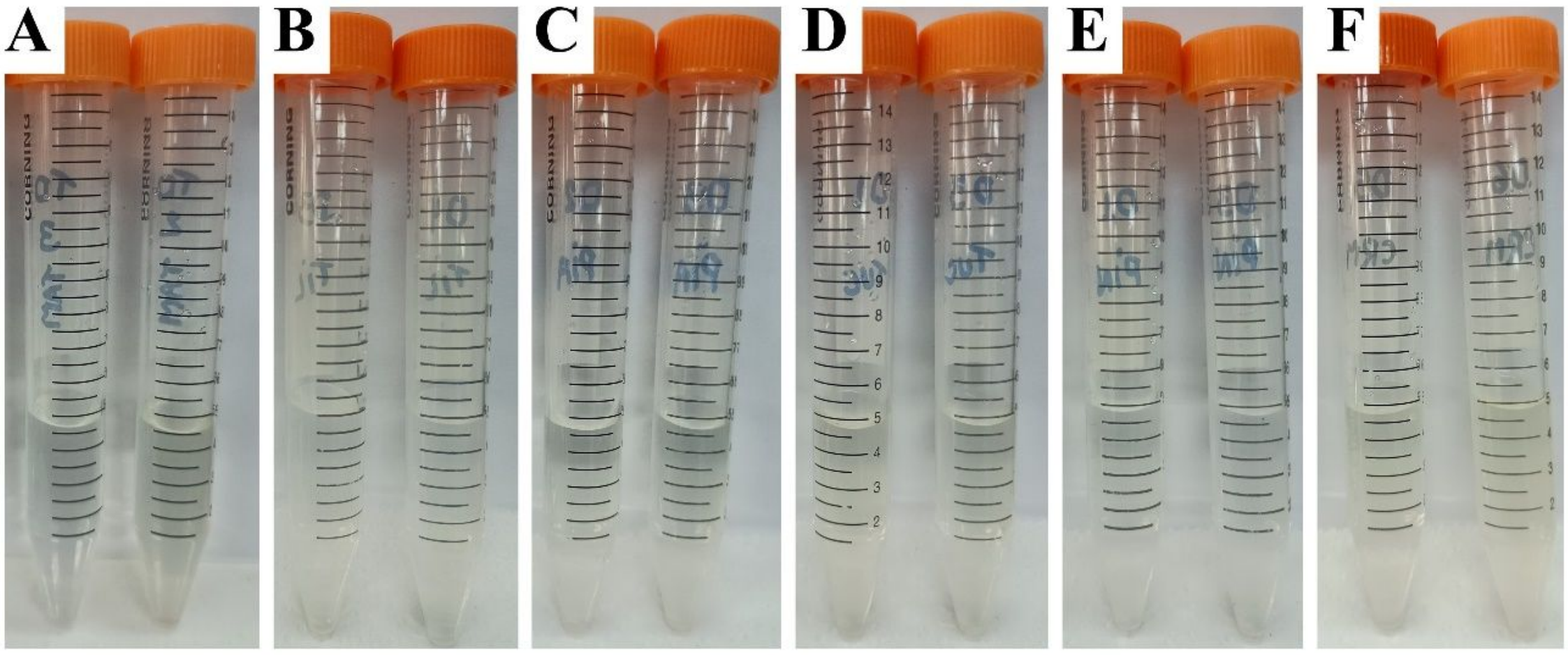

Figure S2. Visual appearance of the digests obtained after AFMAD under optimized conditions for fish tissue samples: **(A)** *Colossoma macropomum*, **(B)** *Oreochromis niloticus*, **(C)** *Megaleporinus conirostris*, **(D)** *Cichla ocellaris*, **(E)** *Pseudoplatystoma corruscans*, and **(F)** cod muscle certified reference material (BCR-422). The formation of clear solutions and the absence of visible particulate residues suggest satisfactory matrix decomposition under fully acid-free conditions.

Table S7. Figures of merit for the proposed acid-free microwave-assisted decomposition method. Internal standards were used to compensate for variations in nebulization efficiency and instrumental drift. The signal intensities of each analyte (except Fe and As) were normalized by the signal intensity of the internal standard (^{209}Bi and ^{89}Y, 5 µg L^{-1}), using the $S_{Analyte}/S_{IS}$ ratio to construct the discovery and quantification curves for the samples.

Analyte	Calibration equation	Linear range (µg L^{-1})	LOD (µg L^{-1})	LOD (mg kg^{-1})[a]	LOQ (µg L^{-1})	LOQ (mg kg^{-1})[a]	R^2
**Fe**	y = 52498x – 7959	0 – 30	0.1	0.06	0.4	0.2	0.994
**Cu**	y = 0.67x - 0.263	0 – 50	0.006	0.02	0.003	0.01	0.997
**Zn**	y = 0.082x + 0.027	0 – 50	0.07	0.2	0.04	0.1	0.990
**As**	y = 2065x -1.077	0 – 50	0.0008	0.003	0.0004	0.001	0.997
**Mn**	y = 0.13x + 0.0007	0 – 5	0.005	0.02	0.002	0.008	0.996
**Cd**	y = 0.076x - 0.0001	0 – 5	0.001	0.004	0.001	0.002	0.999

[a]LOD and LOQ calculated for the original solid sample and a mass of 100 mg

Table S8. Description of the microwave-assisted decomposition procedures used to evaluate green metrics with the AGREEprep software.

	**Parameter**	**Our work – Fish (H_2O_2 decomposition)**	**Sirhan et al. – Fish (acid decomposition)**	**Šteković et al. – Tuna, and Squid (acid decomposition)**	**Singh et al. – Bivalve (acid decomposition)**	**Pothuraju et al. – Fish (acid decomposition)**		
**1.**	**Sample preparation placement**	Ex situ	Ex situ	Ex situ	Ex situ	Ex situ	Ex situ	Ex situ
**2.**	**Hazardous materials**	5 mL	6 mL	5.18 mL	2 mL	9 mL	4 mL	5 mL
**3.**	**Sustainability**	<25%	<25%	<25%	<25%	<25%	<25%	<25%
**4.**	**Waste**	50 mL	50 mL	50 mL	25 mL	100 mL	100 mL	150 mL
**5.**	**Size economy**	100 mg	400 mg	300 mg	500 mg	1000 mg	1000 mg	1000 mg
**6.**	**Sample throughput**	43	21[1]	7[1]	24[1]	15[1]	15[1]	15[1]
**7.**	**Integration and automation**	4 steps: weight, reagent addition, microwave heating program, volume adjustment<br>2. No automation degree	4 steps: weight, reagent addition, microwave heating program, volume adjustment<br>2. No automation degree	6 steps: weight, reagent addition, ultrasonic bath, volume adjustment, microwave heating program, volume adjustment<br>2. No automation degree	4 steps: weight, reagent addition, microwave heating program, volume adjustment<br>2. No automation degree	4 steps: weight, reagent addition, microwave heating program, volume adjustment<br>2. No automation degree	4 steps: weight, reagent addition, microwave heating program, volume adjustment<br>2. No automation degree	4 steps: weight, reagent addition, microwave heating program, volume adjustment<br>2. No automation degree
**8.**	**Energy consumption**	47 Wh	73 Wh	275 Wh	99 Wh	80 Wh	80 Wh	80 Wh
**9.**	**Post-sample preparation**	Advanced MS with high energy and/or noble gas	GC and HPLC with non-MS detection,	Spectrophotometry, surface analysis techniques,	Advanced MS with high energy and/or noble gas	Advanced MS with high energy and/or	Advanced MS with high energy and/or noble gas	Advanced MS with high energy and/or noble gas

	**configuration for analysis**	consumption: ICP-OES, ICP-MS, etc	atomic absorption spectroscopy, capillary electrophoresis, etc.	voltammetry, potentiometry, etc	consumption: ICP-OES, ICP-MS, etc	noble gas consumption: ICP-OES, ICP-MS, etc	consumption: ICP-OES, ICP-MS, etc	consumption: ICP-OES, ICP-MS, etc
**10.**	**Operator's safety**	2 hazards	5 hazards	3 harzards	5 harzards	5 hazards	3 hazards	3 hazards

[1]For methods where the number of simultaneously processed samples was not explicitly reported, the nominal capacity of the microwave digestion system rotor was considered to estimate sample throughput and energy consumption per sample.
